# The Synthesis, Self-Assembled Structures, and Microbicidal Activity of Cationic Gemini Surfactants with Branched Tridecyl Chains

**DOI:** 10.3390/molecules24234380

**Published:** 2019-11-30

**Authors:** Martin Pisárčik, Matúš Pupák, Miloš Lukáč, Ferdinand Devínsky, Lukáš Hubčík, Marián Bukovský, Branislav Horváth

**Affiliations:** 1 Department of Chemical Theory of Drugs, Faculty of Pharmacy, Comenius University, SK-83232 Bratislava, Slovakia; lukac@fpharm.uniba.sk; 2 State Institute for Drug Control, SK-82508 Bratislava, Slovakia; matus.pupak@sukl.sk; 3 Faculty of Pharmacy, Comenius University, 83232 Bratislava, Slovakia; devinsky@fpharm.uniba.sk; 4 Department of Physical Chemistry of Drugs, Faculty of Pharmacy, Comenius University, SK-83232 Bratislava, Slovakia; hubcik@fpharm.uniba.sk; 5 Department of Cell and Molecular Biology of Drugs, Faculty of Pharmacy, Comenius University, SK-83232 Bratislava, Slovakia; bukovsky@fpharm.uniba.sk; 6 NMR Laboratory, Comenius University, SK-83232 Bratislava, Slovakia; horvath@fpharm.uniba.sk

**Keywords:** gemini surfactant, polymethylene spacer, tridecyl chain, zeta potential, microbicidal activity

## Abstract

Cationic gemini surfactants with polymethylene spacer and linear alkyl chains containing an even number of carbon atoms have been extensively studied in the recent past, with the emphasis put on the determination of their aggregation behaviour in aqueous solution and their biological properties. However, the information on the aggregation of branched gemini surfactants with an odd number of carbon atoms in their alkyl chains is only sparsely reported in the literature. To help cover this gap in the research of cationic gemini surfactants, a series of branched bisammonium cationic gemini surfactants with an odd number of carbon atoms in alkyl chains (tridecane-2-yl chains) and a polymethylene spacer with a variable length ranging from 3 to 12 carbon atoms have been synthesized and investigated. Critical micelle concentration, which was determined by three methods, was found to be in the order 10^−4^ mol/L. A comparison of the obtained data of the novel series of tridecyl chain geminis with those of gemini surfactants with dodecyl chains and an identical spacer structure revealed that structural differences between both series of gemini surfactants result in different aggregation and surface properties for surfactants with 6 and 8 methylene groups in the spacer (*N*,*N*’-bis(tridecane-2-yl)-*N*,*N*,*N*’,*N*’-tetramethylhexane-1,6-diaminium dibromide and *N*,*N*’-bis(tridecane-2-yl)-*N*,*N*,*N*’,*N*’-tetramethyloctane-1,8-diaminium dibromide) with the cmc values 8.2 × 10^−4^ mol/L and 6.5 × 10^−4^ mol/L, respectively, as determined by surface tension measurements. Particle size analysis showed the formation of small stable spherical micelles in the interval between 2.8 and 5 nm and with zeta potential around +50 mV, which are independent of surfactant concentration and increase with the increasing spacer length. Microbicidal activity of **13-s-13** gemini surfactants was found to be efficient against Gram-positive, Gram-negative bacteria and yeast.

## 1. Introduction

Gemini cationic bisammonium surfactants with various structural modifications both in the spacer part and in alkyl chains continue to attract broad interest from the scientific community as well as various industrial branches and applications over several decades [1,2]. Recent studies indicate the application of cationic gemini surfactants as efficient tools in retardation during the dyeing process and dye solubilisation [3,4,5], corrosion inhibition [6,7,8,9,10,11,12,13,14], as air entrenching agents for concrete [15,16], in enhanced oil recovery [17,18], lignite [19], and coal pitch [20] moistening, etc. Cationic gemini bisammonium surfactants also play an important role in modern nanotechnologies as modifiers of silica nanosheets [21], dispersants of carbon nanotubes [22], or efficient stabilisers of silver [23,24,25] and gold nanoparticles [26,27]. Due to their molecular structure consisting of two hydrophobic chains and two ammonium polar heads connected with a spacer, aggregation properties of gemini surfactants are superior to those of conventional single-chain surfactants. Micelles of bisamonium gemini surfactants, even those with shorter decyl chains and a polymethylene spacer of 2 to 12 CH_2_ groups in length have cmc values in the decimal order of 10^−4^ mol/L [28], which is far below the cmc value of a single-chain ammonium surfactant with the decyl chain. Aggregation behaviour of gemini molecules can be controlled by the structural modification of the spacer part of a gemini molecule through the insertion of various functional chemical groups, e.g. sulphur, oxygen, *N*-methylated nitrogen, methylene [29,30], hydroxy group [31,32]. To a certain extent, the aggregation of cationic gemini molecules is also affected by the modification of alkyl parts, such as cyclododecyl chains [33] or of the molecule head group [34]. Cationic bisammonium gemini surfactants with simple polymethylene spacer belong to one of the most studied groups of cationic geminis. Their aggregation properties strongly depend on the length of polymethylene spacer, i.e. on the number of methylene groups that compose the spacer [35,36,37,38,39,40]. Depending on this spacer length, gemini molecules aggregate into a broad range of colloidal structures [41]. Double positive charge of bisammonium polar parts of gemini molecules is responsible for strong attractive interactions with various polyions such as sodium hyaluronate [42,43], sodium carboxymethylcellulose [44], sodium polystyrene sulfonate [45], DNA [46,47], and proteins [48]. In the field of nonionic systems, they interact with cyclodextrins [49,50] or block copolymers [51,52]. Bisammonium gemini molecules with the polymethylene spacer have also been found to be an efficient solvation tool for electronically excitable molecules [53]. A lot of effort has been devoted to the investigations of the biological activities of gemini surfactants. Gemini bis(dodecyltrimethylammonium) surfactants with simple polymethylene chains of 3 to 6 CH_2_ groups in the spacer were tested for antifungal [54] and antibacterial activity [55,56] against various environmental strains. Polypropylene nonwovens were modified with gemini surfactants with hexamethylene spacer to acquire biocidal properties [57]. 

Branching or increasing the number of carbon atoms in the side chains of surfactant alkyl tails increases the hydrophobicity [58], tail solvation, and adsorption, as is reported for ethoxylated nonionic and anionic surfactants [58,59]. Improved foaming, detergency, and wetting were observed in various formulations containing surfactants with branched hydrophobic parts [59]. Branched hydrocarbon chains in anionic surfactant molecular structures were found to be useful as replacements for environmentally hazardous fluorocarbon surfactants and polymers [60]. Highly branched anionic surfactants with a prospective use in enhanced oil recovery show low surface energy and are stable at harsh conditions [61]. Studies of aggregation properties of linear or branched surfactants with the odd number of carbon atoms are rare to find in the literature, presumably due to the more complex or financially demanding preparation or availability of odd-chain hydrocarbons for surfactants synthesis. There are very few studies reporting differences in physicochemical characteristics or aggregation behaviour between series of surfactants with even and odd carbon numbers in the alkyl chain. For example, enthalpy of micellization of gemini surfactants is reported to vary for geminis with even and odd numbers of carbon atoms in alkyl chains. Enthalpy values of the surfactants with even numbered alkyl chains vary from endothermic to exothermic with the increasing chain length, whereas the values of the surfactants with odd numbered alkyl chains are all endothermic [62]. 

The purpose of this paper is to report aggregation in the bulk and surface behaviour at the air/water interface of cationic bisammonium gemini surfactants with the odd number of carbon atoms and branched hydrophobic chains and a polymethylene spacer of variable length, which would extend the knowledge of aggregation properties and the biological activity of this group of gemini surfactants. To the best of our knowledge, this kind of gemini surfactant has neither been frequently reported nor studied systematically. Within the framework of this study, synthesis, determination of aggregation properties, and microbicidal activity of tridecane-2-yl bisammonium gemini surfactants with 13 carbon atoms in each of its hydrophobic chains and a polymethylene spacer of variable length, denoted as **13-s-13** where **s** is the number of methylene units in the spacer, are reported. The results are compared with the well-studied group of gemini surfactants alkanediyl-α,ω-bis(*N*-dodecyl-*N*,*N*-dimethylammonium bromides), hereinafter referred to as **12-s-12,** which have 12 carbon atoms in each of their hydrophobic chains and a polymethylene spacer of variable length **s**.

## 2. Materials and Methods 

### 2.1. Synthesis

**Scheme 1:**
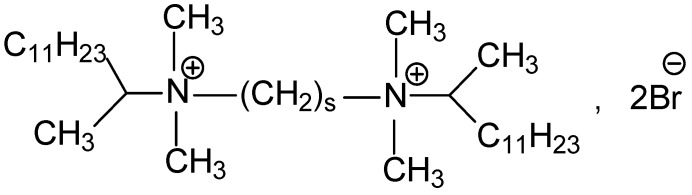
Molecular structure of **13-s-13** gemini surfactants with the spacer number values **s** = 3, 4, 5, 6, 8, 10, 12.

The molecular structure of **13-s-13** gemini surfactants is shown in Scheme 1. 

Gemini surfactants **13-s-13** at each spacer length **s** were prepared by the reaction of 1 eq *N*-tridecane-2-yl-*N,N*-dimethylamine with 0.51 eq α,ω-dibromoalkane in 30 mL of anhydrous acetonitrile under reflux for eight hours. After cooling the mixtures to room temperature, the solvent was evaporated in vacuum and the crude residue was purified by manifold crystallisation from anhydrous acetone. The final products were dried in a vacuum desiccator using P_4_O_10_. 

Tertiary amine was prepared using hydrogenation under the pressure of tridecane-2-one with dimethylamine and platinum as a catalyst. Any other chemicals and solvents that were used were obtained from Fluka, Aldrich, Centralchem, and Merck without further purification. Anhydrous solvents, if used, were prepared using the standard laboratory procedures. The IR spectra were recorded on Nicolet FT IR 6700 spectrophotometer (Thermo Scientific), and the ^1^H and ^13^C NMR spectra were recorded on the Varian Gemini 2000 spectrometer operating at a proton ^1^H NMR frequency of 300 MHz and at a carbon ^13^C NMR frequency of 75.5 MHz. The tetramethylsilane (TMS, 0.01%, *v*/*v*) was used as an internal standard in ^1^H NMR spectra and a peak at 77.16 ppm was used as the reference standard in ^13^C NMR spectra. The FIDs were Fourier transformed. The values of chemical shifts are given in ppm and the values of the interaction constants (*J*) are given in Hz. In the following section, NMR data and IR spectra values for **13-s-13** gemini surfactants are provided.

*N,N’*-bis(tridecane-2-yl)-*N,N,N’,N’*-tetramethylpropane-1,3-diaminium dibromide

Yield: 58%, MP: 215–218 °C. ^1^H NMR: (CDCl_3_, TMS): 0.88 (t, *J* = 7.0 Hz, 6H), 1.20–1.42 (m, 36H), 1.50 (s, 3H) 1.51 (s, 3H), 1.98–2.08 (m, 2H), 2.65–2.78 (m, 2H), 3.33 (s, 6H), 3.37 (s, 6H), 3.57–3.67 (m, 2H), 3.71–3.92 (m, 4H). ^13^C NMR: (CDCl_3_, TMS): 72.7, 57.5, 49.2, 49.0, 31.9, 30.3, 29.6, 29.5, 29.4, 29.3, 27.2, 22.7, 18.6, 14.4, 14.1. IR(ν_max_/cm^−1^): 2954, 2922, 2852, 1494, 1468, 722.

*N,N’*-bis(tridecane-2-yl)-*N,N,N’,N’*-tetramethylbutane-1,4-diaminium dibromide

Yield: 58%, MP: 165–167 °C. ^1^H NMR: (CDCl_3_, TMS): 0.88 (t, *J* = 6.9 Hz, 6H), 1.20–1.5 (m, 36H), 1.48 (s, 3H) 1.50 (s, 3H), 1.82–2.10 (m, 2H), 2.28–2.52 (m, 4H), 2.60–2.78 (m, 2H), 3.19 (s, 6H), 3.23 (s, 6H), 3.47–3.52 (m, 2H), 3.64–3.95 (m, 4H). ^13^C NMR: (CDCl_3_, TMS): 70.6, 61.4, 48.6, 48.2, 31.8, 30.1, 29.5, 29.4, 29.3, 27.1, 22.6, 19.9, 14.2, 14.1. IR(ν_max_/cm^−1^): 2953, 2918, 2850, 1494, 1468, 721.

*N,N’*-bis(tridecane-2-yl)-*N,N,N’,N’*-tetramethylpentane-1,5-diaminium dibromide

Yield: 39.8%, MP: 233–237 °C. ^1^H NMR: (CDCl_3_, TMS): 0.88 (t, *J* = 6.9 Hz, 6H), 1.20–1.5 (m, 36H), 1.44 (s, 3H) 1.46 (s, 3H), 1.59–1.70 (m, 2H), 1.85–1.96 (m, 2H), 2.03–2.20 (m, 4H), 3.28 (s, 6H), 3.30 (s, 6H), 3.58–3.68 (m, 2H), 3.70–3.88 (m, 4H). ^13^C NMR: (CDCl_3_, TMS): 69.3, 62.3, 48.5, 48.3, 31.9, 30.1, 29.6, 29.5, 29.4, 29.3, 27.1, 22.6, 21.8, 14.1(3), 14.1(2). IR(ν_max_/cm^−1^): 2955, 2923, 2853, 1467, 722.

*N,N’*-bis(tridecane-2-yl)-*N,N,N’,N’*-tetramethylhexane-1,6-diaminium dibromide

Yield: 79%, MP: 237 °C. ^1^H NMR: (CDCl_3_, TMS): 0.88 (t, *J* = 6.9 Hz, 6H), 1.20–1.5 (m, 38H, 23× CH_2_), 1.41 (s, 3H) 1.44 (s, 3H), 1.58–1.68 (m, 4H), 1.89–1.94 (m, 2H), 2.00–2.15 (m, 4H), 3.32 (s, 6H), 3.34 (s, 6H), 3.60–3.75 (m, 6H). ^13^C NMR: (CDCl_3_, TMS): 69.1, 62.4, 48.4, 31.9, 30.1, 29.6, 29.54, 29.47, 29.4, 29.3, 27.3, 22.7, 21.3, 14.1, 14.0. IR(ν_max_/cm^−1^): 2953, 2924, 2853, 1468, 722.

*N,N’*-bis(tridecane-2-yl)-*N,N,N’,N’*-tetramethyloctane-1,8-diaminium dibromide

Yield: 66%, MP: 156–157 °C. ^1^H NMR: (CDCl_3_, TMS): 0.88 (t, *J* = 7.0 Hz, 6H), 1.20–1.55 (m, 46H), 1.43 (s, 3H) 1.45 (s, 3H), 1.82–1.96 (m, 6H), 3.28 (s, 6H), 3.29 (s, 6H), 3.62–3.75 (m, 6H). ^13^C NMR: (CDCl_3_, TMS): 68.9, 63.0, 48.5, 48.3, 31.9, 30.0, 29.6, 29.5, 29.4, 29.3, 27.5, 27, 25.4, 22.7, 22.1, 14.1, 14.0. IR(ν_max_/cm^−1^): 2951, 2923, 2853, 1469, 723.

*N,N’*-bis(tridecane-2-yl)-*N,N,N’,N’*-tetramethyldecane-1,10-diaminium dibromide

Yield: 81%, MP: 171–173 °C.^1^H NMR: (CDCl_3_, TMS): 0.88 (t, *J* = 6.7 Hz, 6H), 1.22–1.55 (m, 50H), 1.45 (s, 3H) 1.47 (s, 3H), 1.76–1.96 (m, 6H), 3.28 (s, 12H), 3.58–3.74 (m, 6H). ^13^C NMR: (CDCl_3_, TMS): 69.0, 63.0, 48.5, 31.9, 30.0, 29.6, 29.5, 29.4, 29.3, 28.4, 28.3, 27.0, 26.0, 22.7, 22.5, 14.1, 14.0. IR(ν_max_/cm^−1^): 2951, 2921, 2851, 1467, 722.

*N,N’*-bis(tridecane-2-yl)-*N,N,N’,N’*-tetramethyldodecane-1,12-diaminium dibromide

Yield: 84%, MP: 84–85 °C. ^1^H NMR: (CDCl_3_, TMS): 0.88 (t, *J* = 6.8 Hz, 6H), 1.20–1.50 (m, 54H), 1.45 (s, 3H) 1.46 (s, 3H), 1.70–1.95 (m, 6H), 3.29 (s, 12H), 3.56–3.68 (m, 6H). ^13^C NMR: (CDCl_3_, TMS): 68.9, 62.9, 48.5, 31.9, 30.0, 29.6, 29.5, 29.4, 29.3, 28.9, 22.70, 26.2, 22.6, 14.1, 14.0. IR(ν_max_/cm^−1^): 2922, 2852, 1484, 1465, 722.

### 2.2. Tensiometry

The surface tension of the surfactant solutions was measured using a Kruss K100MK2 tensiometer with the Wilhelmy plate method. The automated dosing of the surfactant stock solution volumes into the solvent in the measurement vessel was realized by a computer-controlled Metrohm 765 Dosimat titrator dosing unit based on a pre-generated table of dosed stock surfactant solution volumes. The automated measurement process was controlled by a Kruss Laboratory Desktop control software. The data were recorded after the surface tension equilibrium value had been reached. The measurements were carried out at 25 °C. The surface excess Π is related to the experimentally determined slope of the surface tension versus log surfactant concentration dependence in the pre-micellar region (d/dlog c)_p_,_T_ using the Gibbs adsorption equation [63]

(1) $$\Pi \;=\;-\frac{1}{2.303\;i\;R\;T}\;{\left(\frac{d\;\gamma }{d\;\log c}\right)}_{p,T}$$

Π is expressed in mol/1000m^2^, (dγ/dlog c)_p_,_T_ is expressed in mN/m, R = 8.31 J K^−1^mol^−1^, and T = 298.15 K. The value of the prefactor i = 3, which applies to ionic gemini surfactants, was used in Equation (1). The value i = 3 is applied for the pre-micellar region of gemini surfactant concentrations where the gemini surfactant is completely ionised in the solution from which adsorption occurs [64]. The area per surfactant molecule A in nm^2^ is calculated from the surface excess Π as follows:

(2) $$A\;=\;\frac{10^{21}}{\Pi \;N_{A}}$$

where N_A_ is the Avogadro’s number. 

### 2.3. Electrical Conductivity

The measurements were performed in a conductivity cell, which was tempered at 25 °C ± 0.1 °C. The stock solution of surfactant in deionised water was prepared in a 100 mL volumetric flask. The conductivity cell was filled with 20 mL of deionized water before the measurement. The stock solution was added to deionized water in the conductivity cell by a computer-controlled Metrohm 765 Dosimat titrator dosing unit based on a pre-generated table of dosed stock surfactant solution volumes. The solution was stirred by a magnetic stirrer during the measurements. The conductivity of the solution was recorded by a conductometer (WTW Tetracon 325) and sent to the computer via the control software. The volume step in the pre-calculated table of dosed volumes of surfactant stock solution was 1 mL for the volumes less than 10 mL, 10 mL for the volumes in the range 10 mL–200 mL, and 200 mL for the dosed volumes larger than 200 mL. The cmc was determined as the break point of two fitting lines in the conductivity versus surfactant concentration dependence using the least squares fitting method. The cmc values with their respective errors were calculated from the fitting constants. The ratio of the slope of the micellar linear region to that of the pre-micellar linear region gives the value of the micelle ionisation degree α [65]. The Gibbs free energy of micellization, ΔG_mic_, is calculated from the cmc and micelle ionisation degree α utilising the following equation, which is valid for dicationic gemini surfactants [66]:

ΔG_mic_ = (3 − 2α) RT ln cmc/55.5 (3)

where α is the micelle ionisation degree, T is the absolute temperature, and R is the gas constant. The constant 55.5 is the number of moles in one litre of solvent (water). 

### 2.4. Fluorescence 

The fluorescence method of critical micelle concentration determination is based on the determination of the intensity ratio of the first and the third vibronic emission peak (I_1_/I_3_) in the pyrene spectrum [67]. This ratio is sensitive to the polarity of the microenvironment surrounding pyrene fluorescence probes, which is represented by the change of the I_1_/I_3_ ratio. It provides two constant levels, one for the pre-micellar region and another for the micellar range of surfactant concentration with the sigmoidal shape of the dependence. Sigmoidal fluorescence profiles were fitted with the Boltzmann sigmoid

(4) $$y\;=\;\frac{A_{1}\;-\;A_{2}}{1\;+\;e^{\left(x\;-\;x_{0}\right)\;/\;\Delta x}}\;+\;A_{2}$$

where the variable y corresponds to the pyrene I_1_/I_3_ ratio value and the independent variable *x* is the gemini surfactant concentration. The constants *A*_1_ and *A*_2_ represent the upper and lower limits of the sigmoid, respectively, and *x*_0_ is the centre of the sigmoid assigned to the cmc. The measurements were performed with a FluoroMax-4 fluorescence spectrometer (Horiba Scientific). The excitation wavelength that was used was 310 nm. Further, 5 μL of pyrene stock solution (0.02 mol/L in methanol) was added to the micellar solutions of the gemini surfactants with the Hamilton syringe. The spectra were recorded at the temperature 25 °C.

### 2.5. Dynamic Light Scattering 

The Brookhaven BI-9000AT digital correlator, BI-200SM goniometer, and an argon laser (wavelength 514.5 nm) were used for dynamic light scattering measurements. The scattered intensity was registered at a constant scattering angle of 90° and at a temperature of 25 °C. Solutions for the light scattering experiments were prepared using deionized water, which was additionally filtered for mechanical impurities through syringe filters with 0.45 µm pore size. To determine the micelle size distributions, a numerical algorithm based on the inverse Laplace transformation was applied to the time correlation function. Micelle diameters were evaluated from the partition size distributions, which were obtained utilising the CONTIN constrained regularisation algorithm [68] with the rejection probability parameter set to 0.5. Due to the large sets of processed data, a custom application software written in Visual Basic was used for automated data format conversion from the measurement files. Five independent measurements and calculations of the time correlation function were carried out for each gemini surfactant at every concentration. The mean value and standard deviation of the mean diameter value for each sample were calculated. 

### 2.6. Zeta Potential 

Zeta potential measurements were performed with the Brookhaven BI ZetaPlus equipment, which is based on the measurement of the electrophoretic mobility utilizing the Doppler frequency shift. Zeta potential values were calculated from the measured mobility using the Smoluchowski limit for the mobility versus zeta potential relationship. The mean value was calculated from a statistical set of 20 zeta potential recordings per each surfactant concentration. The measurements were taken at 25 °C. 

### 2.7. Microbicidal Activity

The microbicidal activity of **13-s-13** gemini surfactants was evaluated in vitro as the minimum microbicidal concentration (MMC). It was determined using the standard broth dilution method [69]. The following representative groups of Gram-positive and Gram-negative bacteria were selected for the experiments: *Staphylococcus aureus ATCC 6538*, *Escherichia coli ATCC 11229*, and *Candida albicans CCM 8186*. Three commercially used quaternary ammonium disinfectants, cetylpyridinium bromide (CPyB), benzyldodecyldimethylammonium bromide (BDDAB, trademark Ajatin), and carbethopendecinium bromide (CPdB, trademark Septonex) were used as the reference microbicidal agents. Their MMC values were determined and the method of testing that was applied within this experimental work is described in the paper [69]. 

## 3. Results and Discussion

### 3.1. Surface Activity

**Figure 1:**
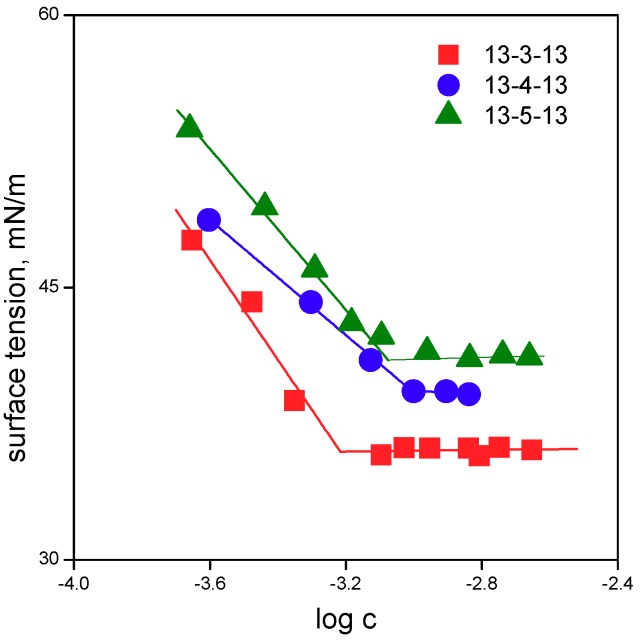
Dependence of surface tension on log surfactant concentration for gemini surfactants **13-s-13** with the spacer length **s** = 3, 4, 5 carbon atoms.

**Figure 2:**
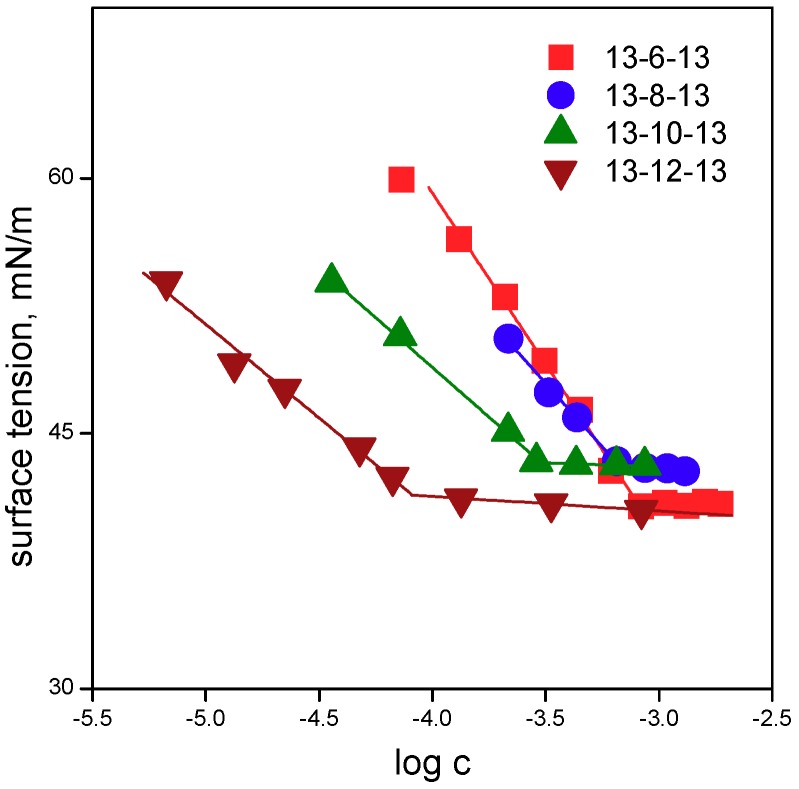
Dependence of surface tension on log surfactant concentration for gemini surfactants **13-s-13** with the spacer length **s** = 6, 8, 10, and 12 carbon atoms.

In Figure 1 and Figure 2, the dependences of surface tension versus log surfactant concentration are shown for gemini surfactant molecules **13-s-13** with tridecyl chains and the spacer length **s** = 3 to 12 carbon atoms. 

**Figure 3:**
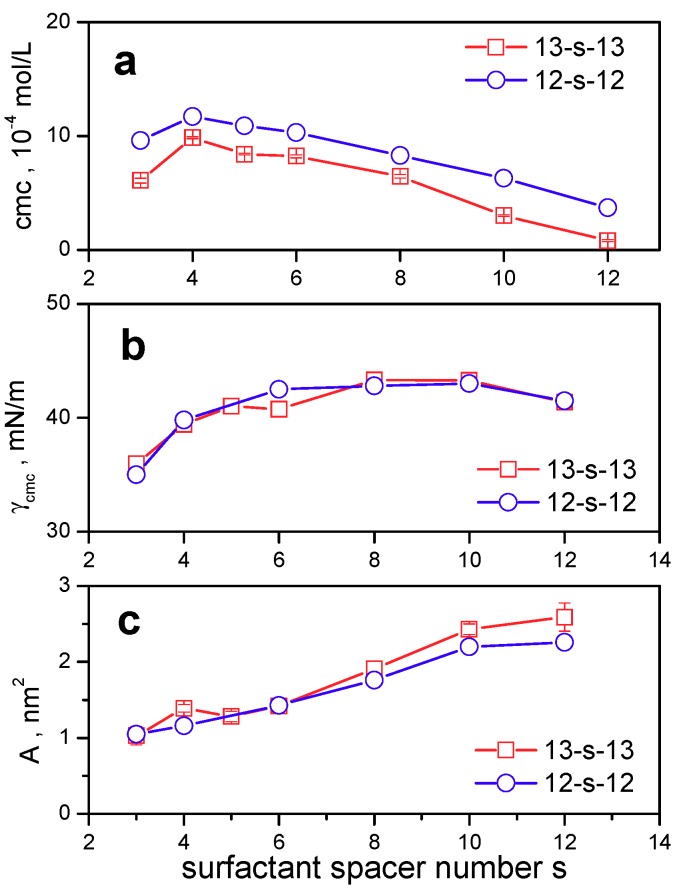
(**a**) cmc, (**b**) surface tension at cmc γ_cmc_, (**c**) area per surfactant molecule at the air/water interface plotted as a function of **13-s-13** gemini surfactant spacer length **s**. Data points that are represented by open circles represent reported surface parameters of gemini surfactants with dodecyl chains **12-s-12**.

Aggregation parameters of **13-s-13** molecules are shown in Figure 3. The cmc values that were determined from surface tension measurements (cmc_γ_ values in Table 1) were calculated as the intersection of the regression lines in the pre-micellar and micellar regions (Figure 1 and Figure 2) and are plotted in Figure 3a. 

Figure 3b,c show the dependence of surface tension at cmc and the area per surfactant molecule, respectively, on the surfactant spacer number **s** for **13-s-13** gemini surfactants. The quantities plotted in Figure 3 are compared with the published values for gemini bisammonium surfactants with dodecyl chains and a polymethylene spacer **12-s-12** [35,36], which are indicated as open circles in the plots of Figure 3. The numerical data for **13-s-13** gemini surfactants are shown in Table 1.

As follows from Figure 3a and Table 1, the cmc dependence on the spacer length s is nonlinear with a maximum at the spacer length **s** = 4. A similar trend in the cmc dependence on the spacer length was found for gemini surfactants with dodecyl chains and polymethylene spacer **12-s-12**. The slight maximum on **13-s-13** cmc dependence is believed to be attributed to the spacer conformational changes. Alkyl chains in molecules of gemini surfactants with polymethylene chain adopt synclinal conformation when the spacer length is up to 4 to 5 CH_2_ groups [35]. As a result, the increased contact between two alkyl chains may lead to a slight increase in the Gibbs free micellization energy and the cmc value. Above the spacer length of 5 CH_2_ groups, a decrease of cmc with the spacer number **s** is observed (Figure 3a). This corresponds with the previous finding of a continuous cmc decrease of **12-s-12** gemini surfactants for spacer values **s** > 5 [35]. The cmc decrease is very moderate in the region of the medium spacer length **s** = 4 to 8. In this region of **s** values, the spacer adopts rigid stretched conformation at the air/water interface at the expense of undesirable contact of the hydrocarbon spacer with bulk water, which increases hydrophobic hydration and restricts micellization, thereby preventing the cmc from further significant decrease [31,70]. At **s** = 10 and 12, the spacer is long enough to show flexible character, which allows its bending into the hydrophobic phase [36]. As a result, a steeper decrease of cmc for **13-10-13** and **13-12-13** gemini surfactants is observed. It is also interesting to note that the shape of the **13-s-13** cmc curve copies the cmc dependence for bisdodecyl gemini surfactants **12-s-12**, showing the maximum at the identical spacer length. However, the **13-s-13** cmc values are lower than those of **12-s-12** at all investigated spacer values [28,71]. This is expectable since the tridecane-2-yl chains of **13-s-13** gemini molecules have one more CH_3_ group than dodecyl chains. Moreover, branched methyl groups of **13-s-13** alkyl chains may also contribute to the more intense hydrophobic intramolecular interactions even though the branching site is located on the carbon atom that is directly attached to the hydrophilic ammonium headgroup.

Values of surface tension at cmc γ_cmc_ (Figure 3b) indicate no significant difference between the surface activity of **13-s-13** and **12-s-12** molecules, which implies no meaningful effect of branched alkyls on the surface activity of bisammonium gemini surfactants with polymethylene spacer. The values for both curves increase up to **s** = 8 and then level off while slightly decreasing at **s** = 12 (Figure 3b). This corresponds with the above described findings, showing decreased hydrophobicity for geminis with medium-sized rigid spacer **s** = 6 to 8, which results in high γ_cmc_. Long and flexible spacer of 12 CH_2_ groups contributes to the hydrophobic interaction between the alkyl chains of gemini molecules, thus allowing γ_cmc_ to decrease with the increasing **s** values.

The values of area per **13-s-13** gemini molecule calculated from the Gibbs adsorption equation (Figure 3c) and plotted as a function of surfactant spacer length **s** in the interval 3 to 12 show three regions. In the first region of small spacer values **s** = 3–5, the interfacial area is low, thus indicating the increased aggregation of gemini molecules with a short spacer. From the data, it can be inferred that the structure and geometry of the head part of **13-s-13** gemini molecule (two ammonium heads and the spacer) is the main factor controlling the interfacial area value of gemini molecules. When the spacer is short, a structural distance d_s_, which corresponds to the extended length of the spacer and is equal to d_s_ = 0.1265(s + 1) in nanometres [72] given that one-half the bond length is assumed at both ends of the spacer, represents the actual spacer length of a short spacered gemini molecule. As a result, molecules are densely arranged at the air/water interface, thus giving small interfacial area values. In our previous study [73], we found that the interfacial area of gemini molecule **12–2–12** with a short spacer is related only to its steric arrangement and is independent of hydrophobic interactions at the interface. In the second region of the spacer values **s** = 6 to 10, the interfacial area increases almost linearly with **s**. The linear increase of the interfacial area with **s** may be proof of the spacer rigidity controlling the behaviour of gemini molecules at the air/water interface and the value of their interfacial area. In the third region of **s** values between 10 and 12 CH_2_ groups, the interfacial area starts to level off, thus indicating the gradual change in the spacer character from rigid to flexible. This conformational change allows the spacer to bend into the hydrophobic air phase, which prevents further interfacial area increase. A study of interfacial properties of **12-s-12** gemini surfactants carried out in the larger interval of **s** values up to **s** = 16 indicates that above **s** = 12, a significant drop in the area values with the increasing spacer length is observed [36]. This, again, emphasises the role of spacer rigidity in the arrangement of gemini molecules at the air/water interface. From the plot in Figure 3c, a slight increase in interfacial area in favour of **13-s-13** surfactants (open squares) is observed. This difference is more pronounced at large **s** values of 10 and 12 carbon atoms in the spacer. This may be a consequence of steric hindrance of branched methyl groups on tridecyl chains, which prevents a denser arrangement of gemini molecules, especially in the case of long flexible spacers **s** = 10, 12. 

### 3.2. Aggregation in Bulk Solution

**Figure 4:**
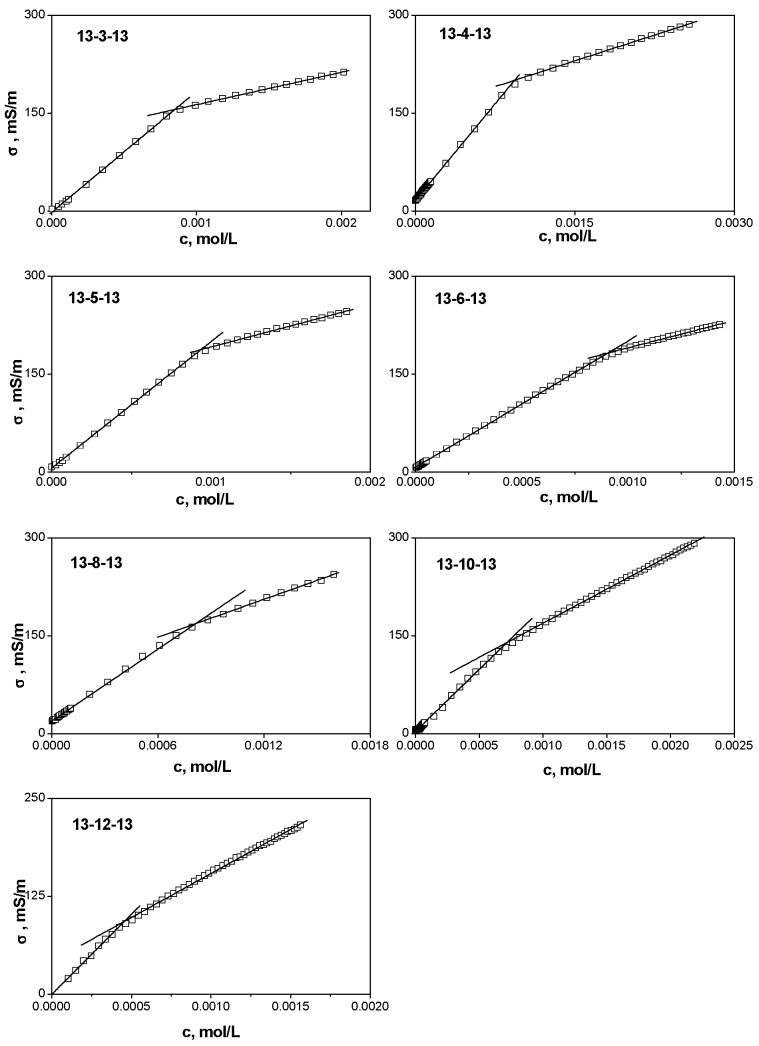
Dependence of electrical conductivity σ on surfactant concentration for gemini surfactants **13-s-13** with the spacer length **s** =3, 4, 5, 6, 8, 10, and 12 carbon atoms.

Dependences of electrical conductivity versus surfactant concentration for **13-s-13** gemini surfactants are shown in Figure 4. The cmc of individual gemini surfactants is determined as the concentration at the intersection of two linear parts in pre-micellar and micellar regions of surfactant concentration c and is calculated from the fitting constants that are obtained by linear regression. 

**Figure 5:**
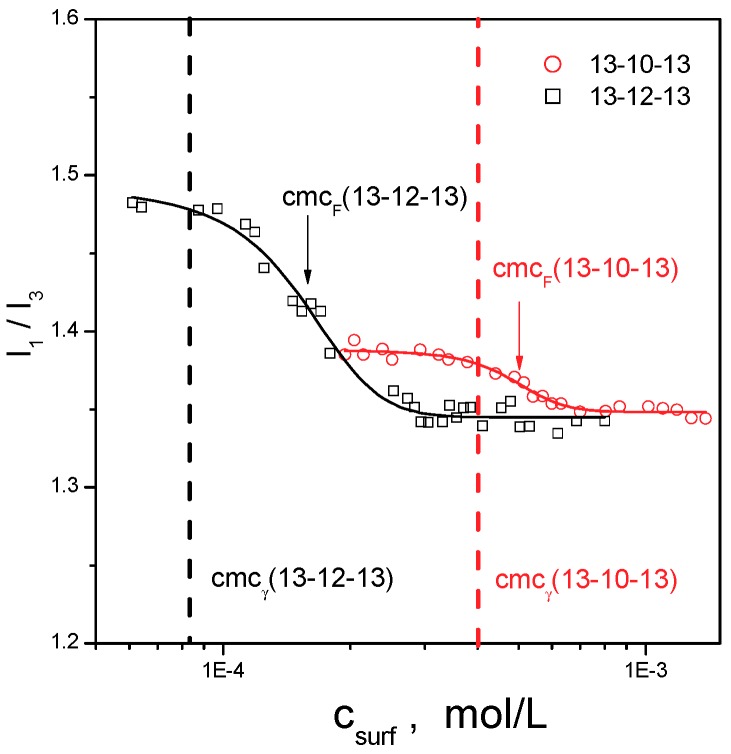
Plot of the pyrene I_1_/I_3_ ratio against surfactant concentration for **13-10-13** and **13-12-13** gemini surfactant aqueous solutions. Dashed lines indicate the cmc determined by surface tension measurements, cmc_γ_.

The degree of micelle ionisation α is calculated as the ratio of the slopes in the micellar and pre-micellar regions. The Gibbs free energy of micellization ΔG_mic_ is calculated from the cmc and micelle ionisation degree based on the Equation (4) of the Material and Methods section. The respective data are summarised in Table 1. The cmc of gemini surfactants was determined by two experimental methods, surface tension and electrical conductivity measurements. The cmc data in Table 1 indicate that cmc_σ_ values are larger for all investigated gemini surfactants than those resulting from surface tension measurements (cmc_γ_). This is to relate to the principle of the conductivity measurements, which require a sufficient number of surfactant ions in a solution to record a detectable change in electrical conductivity. On the other hand, the change in the arrangement of surfactant molecules at the air/water interface as a result of micellization in the solution volume happens almost instantly. In respect to the above, a change in surface tension corresponding to the micellization is observed at a lower surfactant concentration than the change in the slope of electrical conductivity versus surfactant concentration dependence, which explains why cmc_γ_ is smaller than cmc_σ_. To shed some light on the influence of the used experimental method on cmc determination, a third fluorescence method was used for the cmc determination of gemini surfactants with the longest spacers investigated, **13-10-13** and **13-12-13** (Figure 5). The method was based on the determination of the ratio of the first and the third vibronic emission peak (I_1_/I_3_) in the pyrene spectrum, which is different in hydrophobic microenvironment of micellar core and in polar aqueous solvent (see the Materials and Methods section for details). cmc_F_ (the subscript F means the symbol for cmc values determined by the fluorescence method) values are determined as the inflection points at the sigmoidal curves and are listed in Table 2 with other relevant statistical quantities of the non-linear fit. cmc values of both surfactants determined by the surface tension method are indicated with dashed lines.

**Figure 6:**
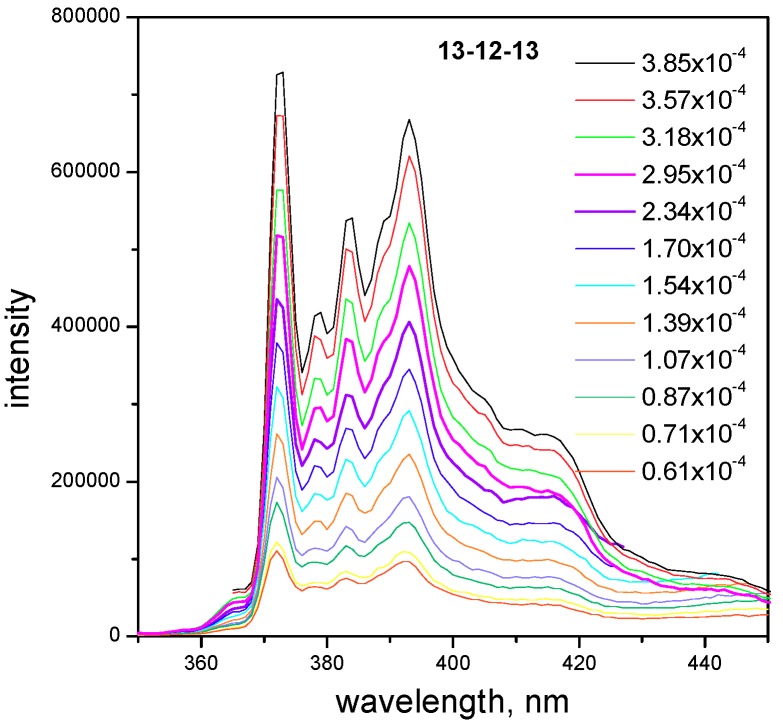
Plot of pyrene fluorescence spectra at selected **13-12-13** gemini surfactant molar concentrations.

As per the results in Figure 5, Table 1 and Table 2, cmc_F_ values for both surfactants are slightly larger than their cmc_γ_ values obtained from surface tension measurements. This difference is within one order of magnitude, which indicates that the fluorescence method is still sensitive enough to register the beginning of micellization. The I_1_/I_3_ ratio in the pyrene fluorescence spectra is changed with the decreasing surfactant concentration where the third vibronic peak intensity is decreasing faster than that of the first one, which results in the increasing I_1_/I_3_ ratio value as the surfactant concentration ist decreased. The plot in Figure 6 shows pyrene fluorescence spectra at selected concentrations for **13-12-13** surfactant as an example (Figure 6). 

When comparing cmcs determined by all three methods in question (cmc data in Table 1 and Table 2), the cmc values follow the pattern cmc_γ_ < cmc_F_ < cmc_σ_. This is also proof of the fact that the micellization process as a phase transition does not take place at a single specific surfactant concentration but rather in an interval of concentration values where the part of smaller surfactant concentrations assigned to the micellization process is covered by surface tension measurements. On the other hand, the less sensitive electrical conductivity method detects the start of surfactant micellization in aqueous solution in the range of larger surfactant concentrations assigned to the micellization process. 

**Figure 7:**
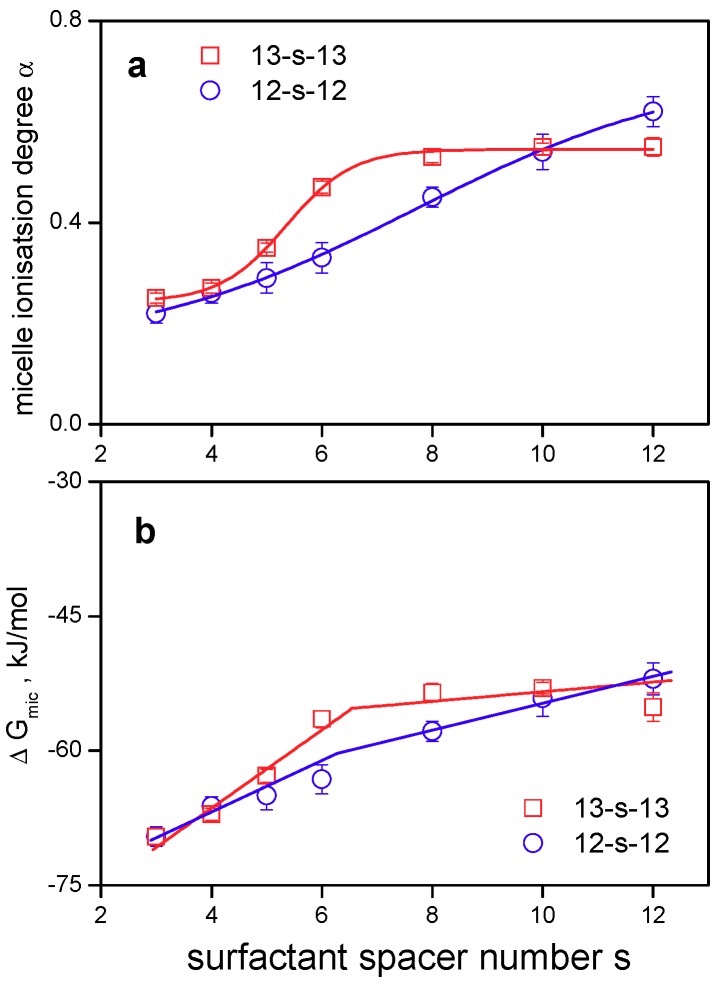
Plot of (**a**) micelle ionisation degree α (**b**) Gibbs free energy of micellization ΔG_mic_ as a function of **13-s-13** gemini surfactant spacer length **s**. Data points represented by open circles show published aggregation parameters [35] of gemini surfactants with dodecyl chains **12-s-12**.

Micelle ionisation degree and the free energy of micellization are increasing with the increasing gemini spacer length **s** (Figure 7a,b, data in Table 1). For comparison purposes, published data for gemini surfactants **12-s-12** with dodecyl chains [35] are also plotted. 

Initially, (Figure 7a) micelle ionisation degree α increases more steeply with surfactant concentration for **13-s-13** surfactants than that of **12-s-12** gemini surfactants and then levels off. The largest difference between α(**13-s-13)** and α(**12-s-12)** values is found in the region of gemini molecules with the medium length spacer **s** = 6, 8. For gemini molecules with a short spacer (**s** = 2) and long spacers (**s** = 10, 12), this difference disappears and the α values are controlled merely by the hydrophobic interactions of alkyl tails with either a very short or a long flexible spacer, as is discussed in detail further in the text related to Gibbs free energy of micellization.

The plot of ΔG_mic_ against the gemini spacer number **s** (Figure 7b) indicates the presence of two straight lines. The first region represented by the spacer length **s** = 2–6 shows a more abrupt increase in ΔG_mic_ corresponding to the larger slope value of dΔG_mic_/ds, which has the meaning of the transfer of single CH_2_ group in the spacer from the bulk to the micellar environment. The value of the Gibbs free energy of this transfer for the spacer values **s** = 2 to 6 and s = 6 to 12, is 4.4 ± 0.7 kJ/mol and 0.5 ± 0.1 kJ/mol, respectively. Micellisation of gemini molecules with a shorter spacer is less spontaneous than that observed for geminis with **s** = 6 and more CH_2_ groups in the spacer, which roughly corresponds with the increase in cmc for gemini surfactants with a short spacer (Figure 3a). On the other hand, the effect of hydrophobic spacer at **s** > 6 results in the decrease in cmc and dΔG_mic_/ds values (Figure 3 and Figure 7), which reflects the increased tendency of these molecules to aggregate into micelles. The comparison of ΔG_mic_ with that of **12-s-12** gemini surfactants, which was calculated from the published α and cmc values [35] (Figure 7b, open circles), provides a similar trend of ΔG_mic_ versus **s** dependence, however with a less steep increase in dΔG_mic_/ds values for **12-s-12** in the pre-micellar region. The largest difference in ΔG_mic_ values between **12-s-12** and **13-s-13** gemini surfactants is observed in the region of spacers with the length 6 to 8 CH_2_ groups. This is a region of rigid spacers which are displaced at the air/water interface or in a micelle shell and do not bend into the micelle hydrophobic core [36]. This difference disappears for very short spacers (**s** = 3) and long spacers (**s** = 10, 12). At the long spacer length, the spacer of gemini molecules is long enough to be bent into the hydrophobic phase (micelle core, air phase at the air/water interface), which enhances the effect of hydrophobic interaction on the values of physical quantities such as α and ΔG_mic_ and the effect of alkyl tail branching has just a minor effect on these physical quantities. In our previous studies, we have shown [38,74] that the geometry of gemini molecules with a very short spacer (**s** = 2, 3 CH_2_ groups) controls their aggregation behaviour, thus rendering the effects of substituents or branching in gemini molecular structure ineffective in terms of the changes in aggregation behaviour of gemini molecules. This is also observed in Figure 7 for both quantities α and ΔG_mic_ where the structural change represented by alkyl branching affects only the aggregation of gemini molecules with medium-length spacers (**s** = 6, 8) with a rigid character. One can conclude that the rigid character of polymethylene spacer in gemini molecules somewhat decreases the level of hydrophobic interaction between the alkyl tails, thus allowing subtle changes in gemini molecular structure such as branching or the presence of small substituents to affect the aggregation properties of gemini molecules.

### 3.3. Size and Zeta Potential of 13-s-13 Micelles

**Figure 8:**
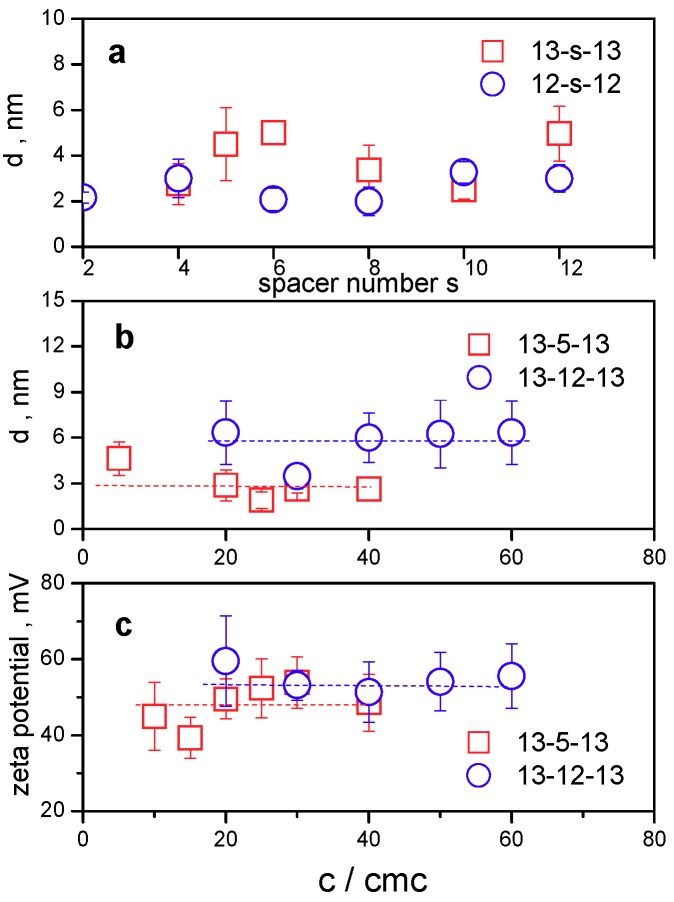
(**a**) Hydrodynamic diameter of **13-s-13** micelles plotted as a function of surfactant spacer number **s**. Data for **12-s-12** micelles from our previous study [38] are represented by open circles. (**b**) Hydrodynamic diameter of **13-3-13** and **13-5-13** micelles plotted as a function of surfactant concentration relative to cmc. (**c**) Zeta potential of **13-3-13** and **13-5-13** micelles plotted as a function of surfactant concentration relative to cmc.

In Figure 8a, the dependence of mean hydrodynamic diameter on surfactant spacer number for all investigated gemini surfactants **13-s-13** is shown. The micelle size was determined for all **13-s-13** gemini surfactants at a constant concentration 20× cmc. 

**Figure 9:**
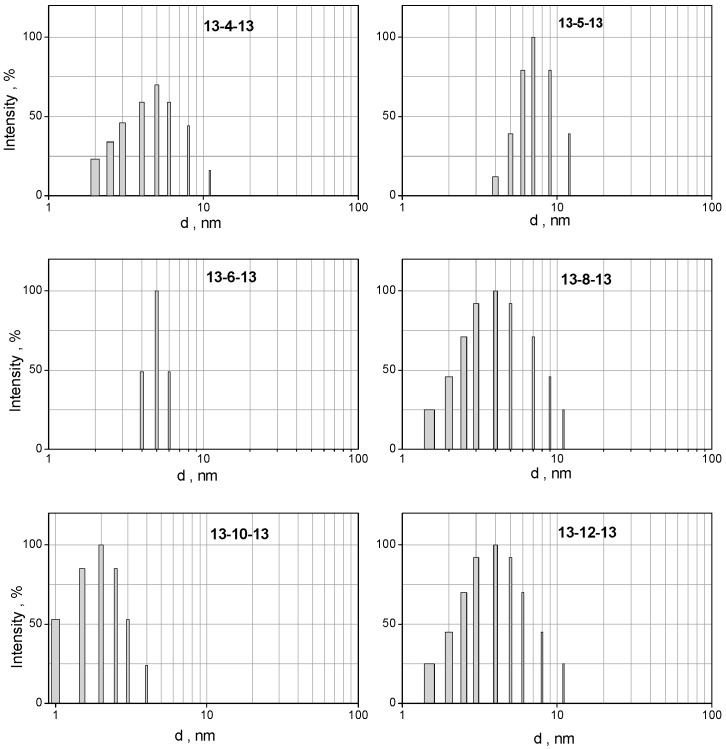
Particle size spectra of **13-s-13** micelles plotted as a function of surfactant spacer number **s**.

The mean size of gemini micelles at all studied spacer number values **s** lies in the range 2.8–5 nm. No significant dependence on the spacer length is observed. However, a slight increase in size is observed for micelles of **13-12-13** gemini surfactant with the longest spacer. These values correspond well to the size of micelles composed of gemini surfactants with dodecyl chains **12-2-12** which have been studied in the literature utilising several experimental methods such as light scattering and small angle neutron scattering. As the neutron scattering studies indicate, **12-s-12** gemini surfactants form ellipsoidal or spheroidal aggregates with a longitudinal diameter of around 4 nm [75]. Slightly smaller diameters were found to be in the range of 1.3–1.8 nm for micelles of gemini surfactants with decyl chains **10-s-10** and the spacer numbers **s** = 6–12 [76]. Even smaller diameter values were obtained for **12-s-12** gemini micelles determined using fluorescence anisotropy (0.86 to 1.24 nm) [53]. This indicates that the values of micelle size are affected to a certain extent by the choice of the experimental method of the size determination. As our study of the **12-s-12** micelle size determination using dynamic light scattering indicates, micelles of **12-s-12** gemini surfactants with **s** = 4, 6, 8, 10, and 12 show particle size between 2 and 3 nm [38]. The diameter values of **12-s-12** micelles are plotted in Figure 8a as open circles. A slight increase in **13-s-13** micelle size at all **s** values may be attributed to the alkyl chain extension by one CH_3_ group. However, this increase seems to not be significant due to the overlap of error bars of both series of gemini surfactants at most spacer length values. Particle size spectra of **13-s-13** micelles (Figure 9) obtained using the CONTIN algorithm show a population of monodisperse small spherical micelles at all investigated spacer length values without the presence of any peaks related to the formation of larger aggregates.

**13-s-13** gemini surfactants form micelles of a constant size, which is documented by the determination of concentration dependence of the hydrodynamic diameter of micelles of two selected gemini surfactants with a medium spacer (**13-5-13**) and a long spacer (**13-12-13**), as it is plotted in Figure 8b. Apart from the size increase between **13-5-13** and **13-12-13** due to the extension of the spacer to 12 carbon atoms and increased spacer flexibility, no significant concentration dependence of micelle size is observed. The average size of **13-5-13** and **13-12-13** micelles indicated by the dashed line in the figure is 2.9 ± 1.0 nm and 5.7 ± 1.2 nm, respectively. This implies that the formation of spherical micelles is stable in size in the studied region of surfactant concentrations. Zeta potential (Figure 8c) of **13-5-13** and **13-12-13** micelles provides positive values independent of surfactant concentration. Again, dashed lines indicate the zeta potential average values of 48.1 ± 5.3 mV and 54.7 ± 3.1 mV for **13-5-13** and **13-12-13**, respectively, which indicates a slight increase in average zeta potential in favour of micelles of gemini molecules with a long spacer. It is interesting to note that **13-s-13** micelles retain their constant size and positive zeta potential over a wide range of surfactant concentrations, even up to the concentration 60×cmc, as resulted from Figure 8b,c. Thisdocuments good stability of **13-s-13** micellar solutions, even at a high surfactant concentration.

### 3.4. Microbicidal Activity

**Figure 10:**
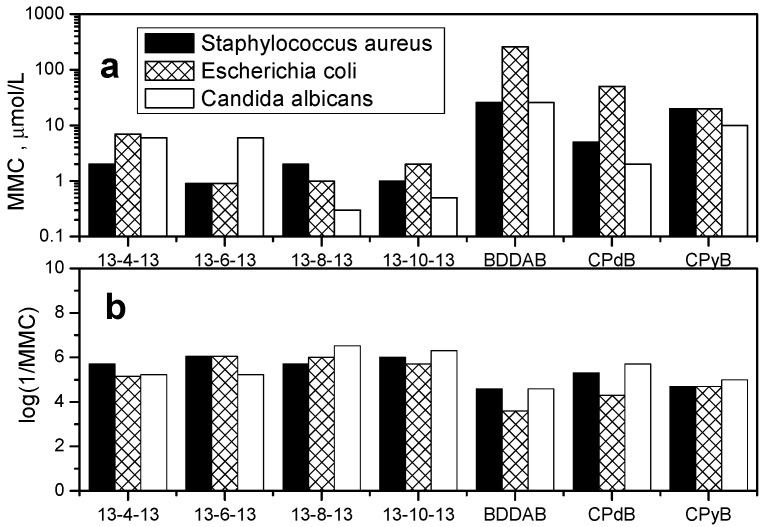
Plot of (**a**) minimum microbicidal concentration (MMC) and (**b**) log (1/MMC) of **13-s-13** gemini surfactants as a function of surfactant spacer length s against Gram-positive bacteria, Gram-negative bacteria, and yeast. The last three data in the plot are MMCs of the reference compounds cetylpyridinium bromide (CPyB), benzyldodecyldimethylammonium bromide (BDDAB), and carbethopendecinium bromide (CPdB).

Microbicidal activity of selected gemini surfactants **13-s-13** was evaluated against Gram-positive bacteria, Gram-negative bacteria, and yeast by determining the minimum microbicidal concentration (MMC) utilizing the diffusion agar technique. The column histograms in Figure 10 show MMC values as well as logarithms of inverse MMC values, with the last three data indicating the microbicidal activity of the reference compounds cetylpyridinium bromide (CPyB), benzyldodecyldimethyl-ammonium bromide (BDDAB, trademark Ajatin), and carbethopendecinium bromide (CPdB, trademark Septonex). 

As the results shown in Figure 10 indicate, all geminis show potent microbicidal activity against the studied bacterial strains and yeast when compared with that of the reference compounds. The activity against Gram-positive *S. aureus* does not show significant dependence on the surfactant spacer number. A slight increase in microbicidal activity with the increasing spacer length is observed against Gram-negative *E. coli* and the yeast *C. albicans*, with the maximum activity in the region of spacer length 8 to 10 CH_2_ groups (Figure 10b). A possible explanation may be related to a more complex composition of cellular membrane of Gram-negative bacteria and yeast considering their double cell wall structure [77,78]. As a consequence, a more hydrophobic gemini molecule is required to efficiently disrupt the complex cell wall structure of this kind, which results in the shift of maximum activity towards longer spacers. However, confirmation of this hypothesis would require the determination of microbicidal activity for gemini molecules with very long hydrophobic spacers **s** > 12, where solubility issues occurred during synthesis and physicochemical measurements. The mechanism of microbicidal action of cationic gemini surfactants is not trivial because it is also affected by other processes such as the sorption of gemini molecules onto microorganisms. According to the study related to the analysis of sorption of cationic, anionic, and nonionic surfactants on biomass composed of Gram-positive, Gram-negative bacteria, and yeast, the highest level of sorption was observed for cationic surfactant adsorbed on the studied microorganisms [79]. This affinity of cationic surfactants to cell membranes relates to the composition of microbial cell walls, which are predominantly negatively charged [80]. Along with the electrostatic interactions facilitating the transfer of cationic surfactant molecules to the vicinity of charged cell membrane, hydrophobic interactions of surfactants with phospholipid bilayer play a crucial role in the initial stage of membrane solubilisation [81]. Experimental studies indicate that the incorporation ability of a surfactant molecule into the phospholipid membrane is mainly governed by the length of the molecule hydrophobic part, which results in a non-linear dependence of microbicidal activity on the alkyl chain length of microbicidally active agent [82]. This interplay of attractive electrostatic and hydrophobic interactions of **13-s-13** gemini molecules with the above described microorganisms may result in their efficient microbicidal action. 

## 4. Conclusions

The present study provides information about the synthesis, physicochemical aggregation, and microbicidal activity of a series of branched bisammonium cationic gemini surfactants **13-s-13** with tridecane-2-yl chains and a variable spacer length. Critical micelle concentration of the novel series of geminis was determined utilising three experimental methods and the mutual differences in cmc values were analysed as a function of the selected method of cmc determination. Micelle ionisation degree and the Gibbs free energy of micellisation were calculated from the electrical conductivity versus surfactant concentration dependencies. A comparison of the obtained data of **13-s-13** gemini surfactants with the corresponding quantities determined for gemini surfactants with dodecyl chains and variable spacer length **12-s-12** reveals that structural differences between the two series of gemini surfactants affect their aggregation in the region of rigid spacers with the medium length of 6 and 8 methylene groups. Particle size analysis utilising dynamic light scattering and zeta potential determination methods show the formation of small stable spherical micelles in the interval between 2.8 and 5 nm and with the positive zeta potential values which are increased with the increasing spacer length. The determination of microbicidal activity of **13-s-13** gemini surfactants revealed efficient action of the geminis against Gram-positive, Gram-negative bacteria, and yeast.

## Figures and Tables

**Table 1:** Surface and aggregation parameters of **13-s-13** gemini surfactants as a function of the spacer length **s**. Values of cmc determined from surface tension measurements **cmc_γ_**, cmc determined from conductivity measurements **cmc_σ_**, surface tension at cmc **γ_cmc_**, area per surfactant molecule **A**, micelle ionisation degree **α**, Gibbs free energy of micellization **ΔG_mic_** are shown as a function of the surfactant spacer number **s**.

Gemini Surfactant	cmc_γ_ 10^−4^ mol/L	cmc_σ_ 10^−4^ mol/L	γ_cmc_ mN/m	A nm^2^	a	ΔG_mic_ kJ/mol
**13-3-13**	6.08 ± 0.21	7.35 ± 0.43	36.0	1.03 ± 0.12	0.25 ± 0.02	−69.6 ± 0.9
**13-4-13**	9.85 ± 0.09	9.20 ± 0.21	39.4	1.39 ± 0.05	0.27 ± 0.02	−67.1 ± 0.7
**13-5-13**	8.41 ± 0.07	9.12 ± 0.38	41.0	1.28 ± 0.07	0.35 ± 0.03	−61.1 ± 0.7
**13-6-13**	8.22 ± 0.13	8.68 ± 0.47	40.8	1.42 ± 0.04	0.47 ± 0.03	−56.5 ± 0.9
**13-8-13**	6.47 ± 0.15	8.13 ± 0.54	43.3	1.91 ± 0.11	0.53 ± 0.01	−53.5 ± 1.0
**13-10-13**	3.01 ± 0.07	7.16 ± 0.31	43.3	2.43 ± 0.07	0.53 ± 0.01	−53.0 ± 0.6
**13-12-13**	0.81 ± 0.07	4.55 ± 0.55	41.4	2.59 ± 0.19	0.55 ± 0.02	−55.1 ± 1.6

**Table 2:** Fitting parameters: n (number o data points) A_1_, A_2_, cmc_F_, dx as obtained from Equation (4) upon fitting the I_1_/I_3_ data to sigmoidal function for gemini surfactants **13-10-13** and **13-12-13**.

Gemini Surfactant	n	A1	A2	cmc_F_ 10^−4^ mol/L	Dx 10^−4^ mol/L
**13-10-13**	24	1.39 ± 0.02	1.35 ± 0.01	4.88 ± 0.14	0.71 ± 0.13
**13-12-13**	30	1.50 ± 0.01	1.34 ± 0.02	1.54 ± 0.06	0.35 ± 0.06
